# Meningitis and sepsis caused by *Streptococcus suis* in an elderly woman: A CARE-compliant case report

**DOI:** 10.1097/MD.0000000000035780

**Published:** 2023-10-27

**Authors:** Kuangyi Li, Shilan Li, Jiana Hong, Weiyin Cheng, Yingjian Zhang, Zhangrong Liang, Qi Tang, Bisheng Shen

**Affiliations:** a Department of Emergency Medicine, Foshan Hospital of Traditional Chinese Medicine, Foshan City, Guangdong Province, China; b The Eighth Clinical Medical College of Guangzhou University of Traditional Chinese Medicine, Foshan City, Guangdong Province, China; c Department of Clinical Nutrition, Foshan Hospital of Traditional Chinese Medicine, Foshan City, Guangdong Province, China.

**Keywords:** meningitis, mNGS, pork, sepsis, *Streptococcus suis*

## Abstract

**Rationale:**

*Streptococcus suis (S suis*)-associated infections are uncommon but life-threatening diseases. The clinical manifestations vary from general symptoms of bacterial infection to fatal meningitis. The clinical manifestation and routine diagnostic testing is not specific enough to obtain well-time diagnosis.

**Patient concerns and diagnosis:**

We report a case of meningitis and sepsis caused by *S suis* infection. A 70-year-old woman presented to our emergency department with generalized pain. After hospital admission, her condition rapidly deteriorated to fever, intracranial hypertension, and disturbance of consciousness. Examination of the blood and cerebrospinal fluid with metagenomic next-generation sequencing and bacterial cultures revealed *S suis* infection.

**Interventions and outcomes:**

After anti-infection therapy with meropenem and vancomycin, the patient recovered and was discharged from the hospital with no residual effects.

**Lessons:**

Human infections with *S suis* are extremely rare. If clinicians encounter a patient with fever, disturbance of consciousness, and intracranial hypertension, especially those who have been exposed to raw pork, *S suis* infection should be considered. Metagenomic next-generation sequencing can be a useful adjunct for the rapid diagnosis of *S suis* infection and aid in the planning of clinical treatment. Meanwhile, public health awareness is necessary to limit the risk of *S suis* infection.

## 1. Introduction

*Streptococcus suis (S suis*) is an ovoid gram-positive anaerobic bacterium that produces capsular polysaccharides, hemolysins, cytokines, and virulence genes that are important for its pathogenicity.^[1]^ It is an emerging pathogen causing bacterial meningitis in Asia.

*S suis* was isolated from the nasal cavity and tonsils of both sick and healthy pigs. However, it spreads naturally between humans and (especially infected) pigs, causing zoonotic infectious diseases.^[2–4]^ *S suis* is divided into 2 types: type I infects only pigs and has no effect on humans, whereas type II infects humans, causing infection at different sites.^[5]^

Human infection with *S suis* has no specific clinical manifestations at the early stage; however, the disease causes it to progress rapidly, leading to meningitis, sepsis, septic shock, arthritis, pneumonia, or endophthalmitis. The clinical manifestations can be divided into 4 categories: common, meningitis or meningoencephalitis, shock, and mixed. The most common type is meningitis or meningoencephalitis, accounting for approximately 50% to 60% of reported cases.^[5–7]^ *S suis* infection can also lead to long-term sequelae, among which hearing impairment is the most common. Approximately 53% of human cases of *S suis* infection combined with meningitis have hearing impairments.^[8]^

Efficacious treatment depends on a rapid and accurate diagnosis. Therefore, early identification of pathogens, diagnosis, and epidemiological investigations are important for saving lives.

Herein, we describe the case of a patient with *S suis* infection who was exposed to raw pork. We reviewed and reported the patient’s medical history, symptoms, etiological detection, and treatment in combination with the relevant literature. We obtained written informed consent from the patient and her family, as well as approval from the Ethics Committee of Foshan Hospital of Traditional Chinese Medicine (Foshan City, China) for publication of this case report.

## 2. Case presentation

A 70-year-old woman presented at our hospital with meningitis and sepsis. The patient was admitted to the emergency department of the Foshan Hospital of Traditional Chinese Medicine with 2-day generalized pain and 1-hour fever. Initially, the patient experienced generalized pain (mainly in the waist, neck, and limbs) without any history of trauma or rheumatic disease, and did not pay attention to it. However, 1 hour before medical attention, she started to show shivering, fever, dizziness, nausea, and vomiting, without limb twitching, slurring of speech, seizures, coma, and cognitive impairment. The patient was then transferred to the emergency department of our hospital.

At the emergency department of our hospital, she presented with a high fever (40 °C), tachycardia (108 bpm), normal blood pressure (122/78 mm Hg), and normal respiratory rate (20 bpm). Physical examination revealed lethargy, poor verbal communication, neck resistance, autonomous activity in the limbs, grade 5 muscle strength in the limbs, increased muscle tone, and a positive Pap sign on the right side. Blood analysis showed a level of high-sensitivity C-reactive protein (136.90 mg/L), serum amyloid A (207.80 mg/L), procalcitonin (1.34 ng/mL), white blood cell count (17.88 × 10^9^/L), and neutrophil percentage (91.70%). Computed tomography of the head revealed no obvious abnormalities. A central nervous system infection and sepsis was suspected, given the clinical feature and laboratory results; therefore, she was admitted to the neurological intensive care unit for further monitoring and treatment.

On the first day of admission, lumbar puncture showed that the cerebrospinal fluid (CSF) was milky white and cloudy, the pressure was 300 mm H_2_O, Pandey test was 3+, white blood cell count was 3587 × 10^6^/L, polynucleated cells accounted for 91%, protein level was 6.749 g/L, chloride ion level was 121 mmol/L, and glucose was <1.0 mmol/L. Blood and CSF samples were collected for metagenomic next-generation sequencing (mNGS) and bacterial culture. Owing to severe illness, she developed respiratory failure after hospitalization. She received tracheal intubation, mechanical ventilation, and meropenem (2 g, q8h) and vancomycin (0.5 g, q8h) as empirical anti-infection therapies. The following day, mNGS of the blood and CSF samples identified *S suis* (blood sequence number: 949; relative abundance: 97.73%; CSF sequence number: 2165678; relative abundance: 99.94%). This result further confirms our conjecture. The treatment was continued using the anti-infection regimen described above. On day-6 of treatment, her symptoms improved and a low fever occurred in the middle of the disease course. Consequently, her body functions gradually recovered. The tracheal catheter was removed and the patient was transferred from the neurological intensive care unit to a general ward for step-down anti-infection treatment. Simultaneously, bacterial cultures of blood and CSF also identified *S suis*, and the drug-sensitivity report showed sensitivity to penicillin, cefotaxime, vancomycin, chloramphenicol, and linezolid but resistance to erythromycin and clindamycin. After more than 3 weeks of systemic treatment, her symptoms gradually improved. Reexamination of infection indicators, blood tests, and CSF testing did not reveal any abnormalities. The patient recovered and was discharged from the hospital. The results of blood analysis and 3 CSF tests after admission to our hospital are shown in Tables 1 and 2.

**Table 1:** The results of blood analysis of the patient after admission.

Date	PCT (ng/mL)	WBC (×10^9^/L)	NEUT%
Day 1 of admission	1.34	17.88	91.7
Day 3 of admission	3.87	18.03	83.2
Day 5 of admission	1.12	12.39	76.3
Day 13 of admission	0.12	21.67	81.5
Day 23 of admission	0.06	7.53	67.2
Reference range	<0.5	3.50–9.50	40.00–75.00

NEUT = neutrophil, PCT = procalcitonin, WBC = white blood cell.

**Table 2:** The results of cerebrospinal fluid of the patient after admission in our hospital.

Date	Property	Pandy-T	C-RBC (×10^6^/L)	C-WBC (×10^6^/L)	Cl-CSF (mmol/L)	Pr-CSF (g/L)	GLU-CSF (mmol/L)
Day 1 of admission	Milky white cloudy with clots	3+	1000	3587	121	6.749	<1.0
Day 5 of admission	Light red cloudy without clots	2+	8000	223	122	3.502	4
Day 14 of admission	Light red cloudy without clots	1+	14,000	37	120	3.639	3
Reference range	–	–	0	0–8	120–132	0.12–0.60	2.5–4.5

Cl-CSF = chlorides in cerebrospinal fluid, C-RBC = red blood cell count, C-WBC = white blood cell count, GLU-CSF = glucose in cerebrospinal fluid, Pr-CSF = protein in cerebrospinal fluid.

Epidemiological investigations showed that the patient had eaten pork before symptom onset; however, specific information was unavailable.

This study was approved (KY[2023]096) by the Ethics Review Board of Foshan Hospital of Traditional Chinese Medicine. Written informed consent was obtained from the patient’s legal guardian for publication of this case report and accompanying data.

## 3. Discussion

*S suis* is regarded as a foodborne pathogen. High-risk groups included humans who had frequent contact with pigs or pork. The risk of *S suis* infection is usually associated with occupation. For example, risk is high for people who breed pigs (e.g., pig farm workers and veterinarians) and occupations related to pork processing, transportation, and sales (e.g., pig slaughterers, workers in processing plants, chefs, transportation drivers, and sales personnel for pig products).^[9]^

According to 1 meta-analysis, among 648 patients infected with *S suis*, 395 (61%) had a history of contact with pigs or pork.^[10]^ A case of suppurative encephalitis caused by *S suis* has been reported in Japan. The patient was a chef with hand wounds, suggesting that the infection was due to contact with raw pork and that bacteria might have entered his body through his hand wounds.^[11]^ According to a retrospective, multicenter study in Catalonia, 9 cases of *S suis* infection were found in 5 hospitals between 2010 and 2019. All patients were male, with an average age of 48 ± 8.9 years, and more than half of them worked on pig farms.^[2]^ In addition, 1 study found that once patients who had undergone splenectomy were infected with *S suis*, their immune system could not rapidly recognize and remove the pathogen owing to impaired immune function. Pathogens accumulates in the body and rapidly destroys tissues, resulting in hypertonic capillaries. Thus, *S suis* infection can lead to severe illness, multiple organ failure, and death.^[7]^ Through epidemiological investigation, we learned that our patient lived in a city and had contact with live pigs 3 days before disease onset, but the infection source was not known.

Since the first case of *S suis* infection in humans was reported in Denmark in 1968, more than 1600 cases of sporadic human *S suis* infection have been reported worldwide. However, there have been 2 major outbreaks of *S suis* infection in humans in China: Jiangsu Province in 1998 and Sichuan Province in 2005.^[12]^ A specific clinical manifestation in the early stage of *S suis* infection is absent, and patients with meningitis usually present with fever, myalgia, neck stiffness, headache, nausea, vomiting, dizziness, and other symptoms.^[13,14]^ The classic meningitis triad is characterized by fever, headache, and an altered state of consciousness.^[15]^ In our patient, the clinical manifestations of fever, generalized pain, headache, dizziness, vomiting, and altered consciousness were consistent with meningitis. The mNGS results of the blood and CSF (as well as the subsequent bacterial culture results) all identified *S suis*, further confirming that the case was meningitis associated with *S suis* infection.

The method of pathogen detection by bacterial culture in the laboratory takes 1 week to complete. Moreover, not all cases of pathogen detection are positive, especially for patients who have been treated with antibiotics at an early stage, and cultures of blood and CSF from such patients may show negative results. Compared to conventional detection methods, mNGS is a more comprehensive, accurate, and rapid method, mainly because it detects the genetic material of the pathogens. The genetic material of *S suis* can also be detected by mNGS in patients who have been previously treated with antibiotics and whose blood cultures are negative.^[7]^

In patients with bacterial meningitis, the risk of death from meningitis associated with *S suis* infection is lower than that for infections caused by other types of pathogenic bacteria such as *Neisseria meningitidis* and *Streptococcus pneumoniae*.^[16]^ Worldwide, the mortality rate of patients infected with *S suis* is approximately 12%; however, in China, it can reach ≤18%.^[8]^ The most common clinical manifestation of *S suis* infection is meningitis, followed by septic arthritis, and approximately half of patients have residual hearing impairment.^[2]^

For the anti-infection regimen of *S suis* infection, β-lactam antibiotics are used empirically to treat *S suis* infections and have been shown to be safe and efficacious.^[17]^ However, for bacterial meningitis without knowledge of the causative pathogen, many guidelines recommend vancomycin plus β-lactam antibiotics.^[18,19]^ In our patient, before the etiological results were obtained, the patient received anti-infection treatment with vancomycin and meropenem with extensive empirical coverage, which showed high efficacy.

## 4. Conclusion

This case report suggests that *S suis* infection should be suspected in patients with fever, disturbance of consciousness, and intracranial hypertension, especially in those exposed to raw pork. Human infection with *S suis* should not be ignored, given that infection with *S suis* can lead to meningitis, sepsis, septic shock, pneumonia, and long-term hearing loss. We should strengthen the health education of high-risk groups, conduct effective science teaching on serious complications and sequelae of *S suis* infection, enhance public health awareness, and strengthen the management of healthy and sick pigs. For example, personnel in the pork-processing industry and chefs should pay attention to protection by wearing gloves at work. If the skin is damaged, they should avoid touching the raw pork (especially those with low immune function) to reduce the risk of *S suis* infection. In addition to the epidemiological characteristics, symptoms, and signs, the diagnosis of *S suis* infection is dependent on the results of blood and CSF cultures. However, for patients with antibiotics, the results of such tests may not be accurate. Therefore, mNGS can rapidly identify pathogens without interfering with antibiotics, which can aid in clinical treatment. However, considering the high cost of mNGS test, it is hard to routinely deploy mNGS test in primary hospitals. Improving the choice of timing to take out such a test of clinicians’ ability is also a challenge.

## Acknowledgments

This work was supported by grants from the High-Level Medical Key Specialty Project in the 14th Five-Year Plan of Foshan City (FSGSPZD145098). The authors sincerely acknowledge the patient and family for their selfless contribution to this report.

## Author contributions

**Conceptualization:** Weiyin Cheng, Yingjian Zhang.

**Data curation:** Zhangrong Liang.

**Formal analysis:** Zhangrong Liang.

**Investigation:** Qi Tang.

**Methodology:** Qi Tang.

**Project administration:** Qi Tang.

**Visualization:** Weiyin Cheng, Yingjian Zhang.

**Writing – original draft:** Kuangyi Li, Shilan Li.

**Writing – review & editing:** Jiana Hong, Bisheng Shen.
